# Screening and brief intervention for problems related to alcohol and other drugs between users of the Family Health Strategy

**DOI:** 10.1186/1940-0640-8-S1-A1

**Published:** 2013-09-04

**Authors:** Angela MM Abreu, Maria Helena N Souza, Rafael T Jomar, Nataly R Queiroz, Daiane B Fernandes

**Affiliations:** 1 Anna Nery School of Nursing, Federal University of Rio de Janeiro, Brazil; 2 National Cancer Institute, Rio de Janeiro, Brazil

## 

The objective of this survey was to identify the lifetime use of alcohol and drugs and the need for implementation of brief intervention for problems related to the use of these substances among users of the Family Health Strategy of the municipality of Rio de Janeiro. A sample of 1031 individuals answered a sociodemographic information form and completed the Alcohol, Smoking, and Substance Involvement Screening Test (ASSIST). Univariate analyses with simple frequency distribution were performed. It was observed that the drugs most used by respondents in life were alcohol and tobacco, and among the most commonly used illicit drugs were marijuana and crack cocaine. Those who needed more brief intervention were tobacco, opioid, hypnotics, marijuana, crack cocaine, and alcohol users. It is important to detect problems associated with alcohol and drug use early in primary care, for this environment promotes health protection and disease prevention.

